# Childhood maltreatment affects depression and anxiety: The mediating role of benign envy and malicious envy

**DOI:** 10.3389/fpsyt.2022.924795

**Published:** 2022-11-16

**Authors:** Xiaojun Li, Linping Tu, Xinsheng Jiang

**Affiliations:** ^1^School of Teacher Education, Nanjing Xiaozhuang University, Nanjing, China; ^2^School of Educational Science, Hunan Normal University, Changsha, China

**Keywords:** childhood maltreatment, depression, anxiety, benign envy, malicious envy

## Abstract

The influence of childhood maltreatment on depression and anxiety has been reported in many studies, and the mechanism of this influence has been described from different perspectives. However, few research has explored the influence of child maltreatment on depression and anxiety from the perspective of benign/malicious envy. Given that, based on social comparison theory, this study explored the mediating effect of benign envy/malicious envy on child maltreatment, depression, and anxiety. The sample of this study consisted of 2,691 Chinese adolescents. The results showed that benign and malicious envy both played a significant role in mediating the relation between childhood maltreatment and depression and anxiety. Interestingly, we found that there were different mechanisms of benign and malicious envy in this relationship, namely, malicious envy promoted the effects of childhood maltreatment on depression and anxiety, whereas benign envy inhibited the effects of childhood maltreatment on depression and anxiety. These findings not only reveal the different mechanisms between the two socially comparable emotions of benign/malicious envy in the early adverse environment and depression and anxiety, but also provide a specific reference for individuals to regulate the depression and anxiety caused by childhood maltreatment and envy.

## Introduction

Today, childhood maltreatment is a major concern around the world and a focus of international child protection (1). Childhood maltreatment is defined as actual or potential harm caused by caregivers or others during childhood, including physical abuse, emotional abuse, sexual abuse, physical neglect, and emotional neglect (2). Several studies have shown that childhood maltreatment affects not only the physical health of an individual (3–6), but can also lead to mental health problems (7–9), such as depression (the main manifestation is low mood) and anxiety (the main manifestation is panic) (10). The relationship between childhood maltreatment and depression and anxiety has been studied in numerous studies. As an example, Wright et al. (11) studied the direct effects of childhood maltreatment on depression and anxiety from an attachment and cognitive perspective; Huh et al. (12) explored the underlying mechanisms of childhood maltreatment and depression and anxiety from the perspective of emotion regulation. In spite of this, few studies have investigated the influence mechanism of childhood maltreatment on these two typical mental health problems from the perspective of social comparison. In the process of upward social comparison, that is, comparison with others who are better than oneself, the individual develops the negative emotion of envy (13). Moreover, depending on perceptions and motivations, envy under social comparison can be classified as either benign or malicious (14–16), and these two types may have different impacts on depression and anxiety. In view of this, this study will explore the mediating role of benign and malicious envy between childhood maltreatment and depression and anxiety based on social comparison theory, in order to provide a theoretical basis for interventions to promote individuals' mental health and reduce their negative emotions.

The link between childhood maltreatment and mental health has been established in previous studies (17, 18). Depression and anxiety, as two typical mental health problems, have also been shown to be influenced by this early adverse environment. For example, Li et al. (19) used the meta-analysis revealed that that individuals abused early in their lives were more likely to have depressive and anxious tendencies; Fergusson et al. (20) demonstrated through a longitudinal study that individuals with early experiences of abuse exhibit stronger tendencies toward depression and anxiety; In a clinical study, Heim and Nemeroff (21) found that childhood maltreatment can lead to partial neurological overactivity, which may increase an individual's risk of developing depression or anxiety disorders. Additionally, previous studies have demonstrated the relationship between childhood maltreatment and depression and anxiety from various perspectives. According to Bernet and Stein (22), abuse during childhood predicts the probability of adult depression; Nanni et al. (23), in a clinical study, found that depressed individuals who suffered abuse in childhood had relapses and remained depressed longer. Furthermore, research has shown that childhood maltreatment makes individuals more prone to anxiety (24, 25). Based on above, we propose the first hypothesis of this study **(H1)**: childhood maltreatment is positively related to depression and anxiety.

Furthermore, from the perspective of social comparison, benign and malicious envy may function as mediators between depression and anxiety. According to the Social Comparison Theory (SCT), individuals have an innate tendency to compare themselves to others (26). Depending on the object of comparison, these comparisons can be categorized as upward or downward social comparisons (27, 28). Individuals who have experienced childhood maltreatment tend to be insecure and more concerned with what others have that they do not (29), and thus more prone to upward social comparison. In upward social comparisons, individuals are prone to the negative emotion of envy (13). In previous research, it has been shown that envy, as a negative emotion generated by social comparison, can lead to a wide variety of mental health disorders in individuals (30). Moreover, studies have shown that envy can be divided into benign envy and malicious envy (14–16), and these two kinds of envy may have different effects on individual mental health. Based on previous research, it has been found that individuals who develop benign envy in upward social comparisons are hopeful, more likely to have relatively positive cognitive styles and emotional experiences, and tend to self-improve in comparisons with others to achieve their desired goals, believing that they can work hard to get what the envied person has (31), so that they are less likely to fall into depression and anxiety. In contrast, individuals who are maliciously envy psychologically perceive themselves as unable to achieve desired goals through self-improvement, while they tend to be destructive and intent on preventing the success of others (32) and reinforce this behavioral tendency through constant social comparison, leading to higher levels of depression and anxiety. Therefore, benign envy may lead to less depression and anxiety, while malicious envy may lead to more depression and anxiety. In addition, previous research using university students has also found that childhood maltreatment is associated with both benign and malicious envy. For example, Zhao et al. (33) found that childhood maltreatment positively predicted malicious envy and negatively predicted benign envy; Li and Xiang (34) explored the mechanisms of influence of childhood maltreatment on benign and malicious envy based on the framework of learned helplessness theory; He and Xiang (35) also confirmed that there is a relationship between childhood maltreatment and benign and malicious envy. Thus, from a social comparison perspective, childhood maltreatment may also affect benign and malicious envy. Individuals who have experienced childhood maltreatment are prone to distrust the outside world and are more willing to choose destructive ways to destroy others' victories in social comparison to achieve their success. As a result, they are more prone to malicious envy than benign envy, which leads to more depression and anxiety. Give that, we suggest a second hypothesis for this study **(H2)**: Benign and malicious envy may play a mediating role between childhood maltreatment and depression and anxiety. And, on the basis of mediating effect, we further hypothesize that: On the one hand, childhood maltreatment can aggravate an individual's tendency to depression and anxiety by increasing malicious envy. On the other hand, it can aggravate an individual's tendency to depression and anxiety by suppressing benign envy.

In summary, this study aims to examine the impact of childhood maltreatment on depression and anxiety from a social comparison perspective and the mediating role that benign and malicious envy plays between them. To this end, we propose two research hypotheses. **H1**: Childhood maltreatment is positively correlated with depression and anxiety; and **H2**: Benign envy and malicious envy mediate the relationship between childhood maltreatment and depression and anxiety, meaning that childhood maltreatment can increase depression and anxiety by increasing malicious envy, as well as by decreasing benign envy.

## Method

### Participants

This study was conducted in two elementary schools, two middle schools and one high school in a coastal city using cluster sampling. The total number of questionnaires distributed was 2,749. After excluding incomplete and apparently random questionnaires, 2,691 questionnaires were returned. Grade characteristics of the subjects were: 1,303 primary school students (grades 4–6), 673 junior high school students (grades 7–8) and 715 high school students (grade 10); gender characteristics were: 1,504 male students and 1,187 female students. The participants ranged in age from 9 to 17 years, with a mean age of 12.50 ± 2.00 years. Detailed participants demographic data can be found in Table 1. After detailed instruction, participants independently completed a series of questionnaires, which included, Childhood Trauma Questionnaire, Benign and Malicious Envy Scale, and Depression Anxiety Stress Scales. The study was approved by the ethics committee of Hunan Normal University (2021, No. 076). The consent procedures followed were consistent with those mentioned in the study protocol submitted for ethical approval. The consent process followed by the Institute is set out below. Firstly, in view of the young age of the participants, the informed consent was communicated to the guardians of the children studying at school, i.e., the teachers concerned, and the parents were informed verbally through the teachers. Therefore, informed consent was obtained from teachers and parents for this study. Secondly, prior to completing the questionnaire, all participants were fully informed of their right to participate in the study, including voluntary participation and withdrawal at any time, and that participation was not associated with merit assessments. Therefore, informed consent was obtained from the participants.

**Table 1:** Demographic data of the sample population (*N* = 2,691).

**Grade**	**Sample**			**Age (years)**	
	**Total**	**Male**	**Female**	**Male**	**Female**
Grade 4	390	195	195	9.91 ± 0.68	9.77 ± 0.65
Grade 5	496	295	201	10.84 ± 0.68	10.72 ± 0.61
Grade 6	417	222	195	11.69 ± 0.65	11.60 ± 0.61
Grade 7	327	179	148	12.65 ± 0.62	12.47 ± 0.60
Grade 8	346	201	145	13.59 ± 0.63	13.39 ± 0.64
Grade 10	715	412	303	15.15 ± 0.60	15.06 ± 0.55

Age are expressed as means ± standard deviations.

### Measures

#### Childhood maltreatment

Childhood Trauma Questionnaire (CTQ) was developed by Bernstein et al. (2) and subsequently revised by Xiang et al. (36). Considering the sensitivity of sexual abuse in the Chinese culture, the study used a revised version of Xiang et al. (36), which removed five items associated with the sexual abuse subscale, leaving 23 items divided into four dimensions, including emotional abuse, physical abuse, emotional neglect, and physical neglect. Each subscale contains five items and an additional three items are set as validity evaluation. The four subscales' Cronbach's alpha coefficients were as follows: 0.69 for emotional abuse, 0.77 for physical abuse, 0.79 for emotional neglect, and 0.61 for physical neglect in the present study. An example of an item is: someone in my family called me “stupid,” “lazy” or “ugly”. All items are scored by a five-point Likert-type scale, ranging from 1 “never” to 5 “always.” The scale has been shown to be appropriate for upper elementary, middle and high school samples (35). The full scale (excluding the sexual abuse subscale) had a Cronbach's alpha of 0.898 in this study.

#### Benign envy and malicious envy

This study used the Benign and Malicious Envy Scale (BeMaS) developed by Lange and Crusius (37), which consists of two scales, Benign Envy and Malicious Envy, each consisting of five items, all items were rated on a six-point Likert scale from 1 “strongly disagree” to 6 “strongly agree.” Example of an item: I want people who are better than me to lose their competitive edge. The scales has been validated for upper elementary and middle and high school students (35), and in this study the Cronbach's alpha was 0.737 for the Benign Envy subscale and 0.786 for the Malicious Envy subscale.

#### Depression and anxiety

The Depression Anxiety Stress Scales (DASS) were developed by Lovibond and Lovibond (38) and adapted by Gong et al. (39). Only two of the subscales, depression and anxiety, were used in this study, comprising a total of 14 items. All items were rated on a four-point Likert scale, with 1 representing “never” and 4 representing “always,” and the higher the score (up to 4), the greater the degree of anxiety or depression. In this study, Cronbach's alpha for the depression and anxiety dimensions were 0.867 and 0.810, respectively. Due to the fact that the scale has only been used in middle and high schools and adult samples previously (40), its reliability in upper elementary grades was separately examined in this study. The reliability coefficients for depression and anxiety were 0.859 and 0.811, respectively, for the upper elementary students.

### Statistical analysis

The data for this study was analyzed using SPSS24.0 and AMOS24.0 software. Firstly, we performed a descriptive analysis using SPSS 24.0 to measure correlations between the potential variables based on the hypotheses of this study. Secondly, a measurement model was constructed using AMOS 24.0 to examine the predictive effect of the observed variables on the latent variables. On this basis, the structural equation model is further constructed. For testing the fit of the model, the Comparative Fit Index (CFI), Standardized Root Mean Square Residual (SRMR) and Root Mean Square Error of Approximation (RMSEA) were used (41). Finally, we used a bias-corrected percentile Bootstrap method with a sample size of 2,000 times and a confidence interval of 95% to examine the mediating effect of benign envy/malicious envy between childhood maltreatment and depression and anxiety. In addition, it is important to note that in the structural equation modeling construct, the observed variables of benign envy/malicious envy, depression and anxiety are each packaged as two variables according to Little et al. (42), which takes into account item balance.

## Results

### Measurement model

The measurement model consists of five potential variables: childhood maltreatment, benign envy, malicious envy, depression and anxiety. The results show that the measurement model showed a good fitness: χ^2^_(44, 2691)_ = 495.075, *p* < 0.001; RMSEA = 0.062; SRMR = 0.0387; CFI = 0.973.

Meanwhile, Table 2 presents the mean (M), standard deviation (SD) and the correlation between the two variables. The results revealed that all variables studied were significantly correlated with each other, except for benign envy, which was not significantly correlated with malicious envy.

**Table 2:** Descriptive statistics and bivariate correlations for all measures.

	**M**	***SD***	**1**	**2**	**3**	**4**	**5**
1. Childhood maltreatment	32.87	11.89	1.00				
2. Benign envy	22.46	4.75	−0.24***	1.00			
3. Malicious envy	11.95	5.49	0.31***	−0.003	1.00		
4. Depression	10.95	3.97	0.53***	−0.27***	0.35***	1.00	
5. Anxiety	11.56	3.81	0.50***	−0.16***	0.33***	0.78***	1.00

^***^ p < 0.001.

### Structural model

**Figure 1:**
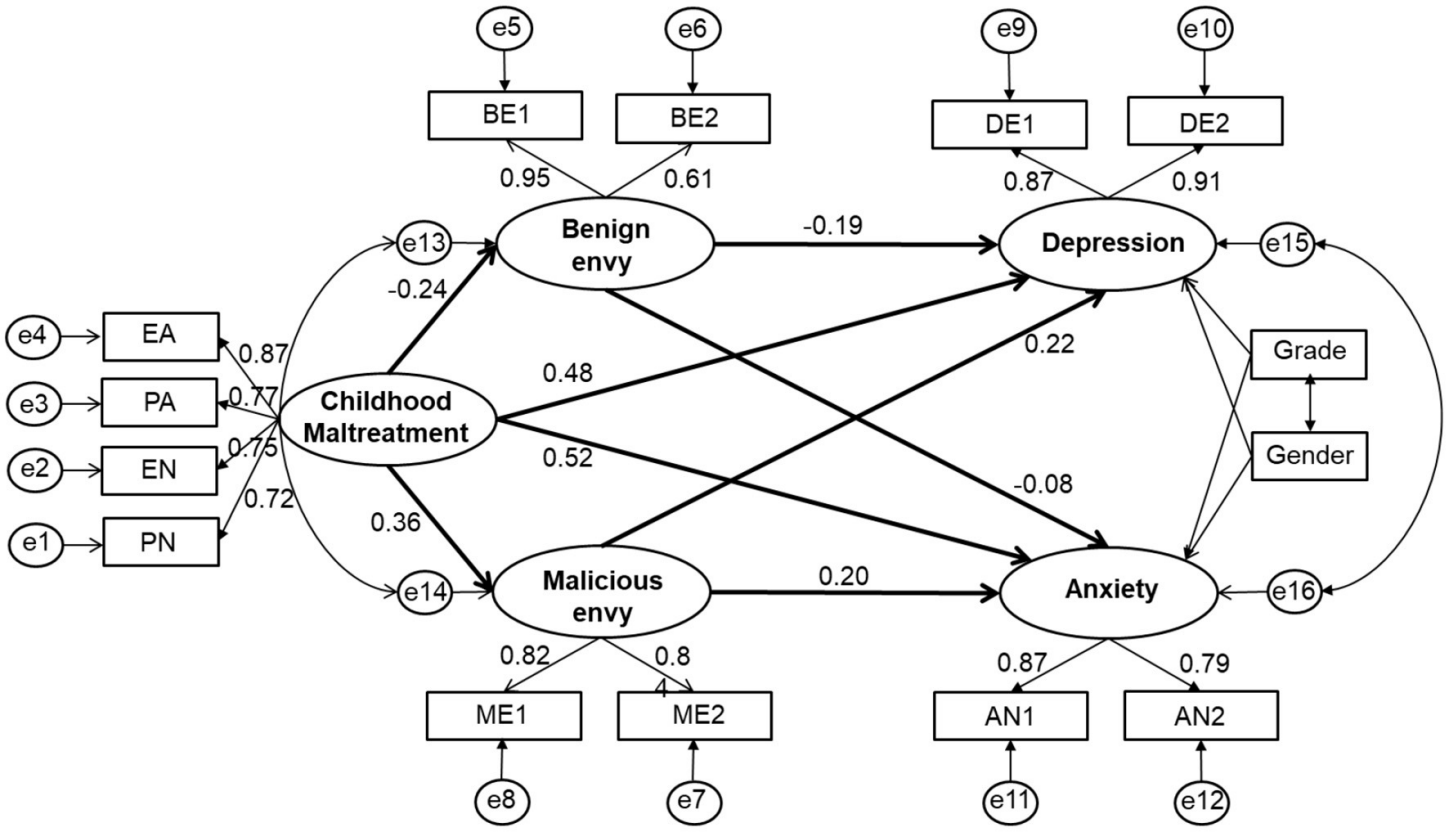
The mediation model. Factor loadings are standardized. EA, PA, EN, PN are the four observed variables of childhood maltreatment (CM). BE1 and BE2 are the two observation variables of benign envy (BE), and ME1 and ME2 are the two observed variables of malicious envy (ME). DE1 and DE2 are the two observed variables of depression (DE), and AN1 and AN2 are the two observed variables of anxiety (AN).

In the absence of benign and malicious envy as mediating variables, childhood maltreatment predicts depression (β = 0.18, *p* < 0.001) and anxiety (β = 0.16, *p* < 0.001). Based on this, model 1 was constructed based on the research hypothesis. By controlling for grade and gender in this model, childhood maltreatment directly and positively predicted depression and anxiety, while childhood maltreatment also positively predicted depression and anxiety by decreasing benign envy and positively predicted depression and anxiety by increasing malicious envy (Figure 1). The results showed that the fitting degree of model 1 was good [χ^2^_(64)_ = 898.359, *p* < 0.001; RMSEA = 0.070; SRMR = 0.039; CFI = 0.952] (Table 3).

**Table 3:** Fit indices of model 1.

	***χ^2^***	***df***	**CFI**	**RMSEA**	**SRMR**	**GFI**	**AGFI**	**TLI**
Model 1	898.359	64	0.952	0.070	0.039	0.953	0.923	0.932

RMSEA, root-mean-square error of approximation; SRMR, standardized root means square residual; CFI, comparative ft index; AIC, Akaike information criterion; ECVI, expected cross-validation index.

### Mediation variable significance

This study used the Bootstrap method to test the significance of the mediating role of benign and malicious envy. We investigated 2,000 bootstraps from the original data, and determined that benign envy mediated the relationship between the impact of childhood maltreatment on depression [95% CI (0.01, 0.03)] and anxiety [95% CI (0.004, 0.02)]. Moreover, childhood maltreatment was associated with depression [95% CI (0.03, 0.05)] and anxiety [95% CI (0.03, 0.05)] through malicious envy (Table 4).

**Table 4:** Standardized indirect effects and 95% confidence intervals.

**Pathways**	**Estimate**	**Lower**	**Upper**	**Effect size (%)**
CM → BE → DE	0.022	0.010	0.032	3.56
CM → BE → AN	0.010	0.004	0.018	1.62
CM → ME → DE	0.039	0.029	0.051	6.31
CM → ME → AN	0.039	0.030	0.050	6.31

CM, childhood maltreatment; BE, benign envy; ME, malicious envy; DE, depression; AN, anxiety.

## Discussion

Based on the social comparison theory, this study examined how childhood maltreatment affects depression and anxiety, as well as the mediating role played by benign and malicious envy. Our results indicated that benign envy inhibited the negative effects of childhood maltreatment on both negative emotions, whereas malicious envy facilitated this relationship mechanism. These findings not only have important theoretical support for the interventions that aim to improve mental health of individuals, but also provide an empirical foundation for practical activities to prevent negative emotions in individuals such as anxiety and depression.

First of all, the results of the study tested hypothesis 1, which suggests that childhood maltreatment positively predicts depression and anxiety. In fact, previous studies using adults as subjects have shown that childhood maltreatment is associated with persistent negative effects on mental health. For example, Springer et al. (10) showed that individuals who were physically abused in childhood displayed more mental health problems in middle age; Thoma et al.'s (43) involving older adults indicated that childhood maltreatment had a negative impact on individuals' mental health throughout their lifetime. Similarly, the study found that childhood maltreatment affected individuals' psychological wellbeing, indicating that childhood maltreatment increased depression and anxiety among adolescents. Moreover, the findings are consistent with those of previous studies involving adults, including Brown et al.'s (44) findings demonstrating that child maltreatment is a significant predictor of depression and anxiety symptoms in adults. The findings of this study, which used adolescent groups as subjects, actually provide further evidence that childhood maltreatment has a negative impact on depression and anxiety in individuals across generations and in a stable manner. And, this result can also be explained by social comparison theory. Individuals who have experienced childhood maltreatment tend to feel more insecure (45) and are prone to envy in social comparisons of what others have that they do not have, leading to depression and anxiety. As a result, individuals who are abused early on are more likely to develop depression and anxiety.

Furthermore, consistent with Hypothesis 2, the results indicate that benign envy and malicious envy play an important mediating role between childhood maltreatment and depression and anxiety, but that there are different mechanisms of action in this relationship. In the case of malicious envy, childhood maltreatment increased malicious envy and consequently depression and anxiety in individuals, and this finding is congruent with previous research. As He et al. (35) found that adolescents who suffered childhood maltreatment had higher levels of malicious envy, which, as a negative emotion, can potentially lead to an individual developing psychological symptoms such as obsessions, fears, depression, and anxiety (46). The present study with adolescents as subjects further confirms that malicious envy plays an important mediating role between childhood maltreatment and depression and anxiety. Moreover, the results of this study are consistent with social comparison theory. From the perspective of social comparison theory, individuals who feel maliciously envy are often prone to a negative state of fear of failure due to their unfavorable social comparison (47), which makes them more likely to develop depression and anxiety. Therefore, individuals who have experienced abuse during childhood are more vulnerable to developing malicious envy, which can lead to depression and anxiety.

In the case of benign envy, however, the study found a completely different mechanism from malicious envy, in that benign envy suppressed the effect of childhood maltreatment on depression and anxiety. As previous research has found, individuals who have suffered childhood maltreatment hold a negative perception of life (34, 48) and are more likely to develop malicious envy than benign envy (33). The present study found that individuals who were less abused in childhood tend to develop benign envy and are thus less likely to fall into depression and anxiety. The reason might be due to the fact that individuals with benign envy demonstrate relatively positive cognitive styles (15), behave more positively when social comparisons occur, believe that the gap between them and the envied person can be closed through self-improvement, and are optimistic about life (49), and thus are less likely to become depressed or anxious. As a result, individuals who experience childhood maltreatment are likely to develop less benign envy, and are more prone to depression and anxiety.

However, there are some limitations to this study. In the construct model, we used depression and anxiety as a single variable to explain the underlying mechanisms of depression and anxiety, which may have caused some bias in the results. Therefore, the role of benign and malicious envy in the relationship between childhood maltreatment and depression and anxiety remains to be studied, and further research should consider the different mechanisms of depression and anxiety.

In summary, this study examines the mechanisms of how childhood maltreatment affects depression and anxiety from the perspective of social comparison theory. While previous studies have examined the effects of childhood maltreatment on individuals' negative emotions from a positive psychology and Big Five personality perspective (46, 50), this study expands the understanding of the mechanisms of childhood maltreatment on negative emotions under a social comparison theoretical perspective. Interestingly, the two types of envy, benign and malicious, play different roles in this mechanism. These results not only contribute to the theoretical study of the effects of childhood abuse on an individual's mental health, but also provide some reference for individuals to regulate depression and anxiety caused by childhood maltreatment and envy.

## Data availability statement

The raw data supporting the conclusions of this article will be made available by the authors, without undue reservation.

## Ethics statement

This research was approved by the scientific research project of the Medical Ethics Committee of Hunan Normal University. The project is titled “The Neural Associations of Benign/Malicious Envy and the Mechanism of Mindfulness Intervention”. The consent procedures followed were consistent with those mentioned in the study protocol submitted for ethical approval. Written informed consent to participate in this study was provided by the participants' legal guardian/next of kin.

## Author contributions

XJ and XL: data collection. XL and LT: data analysis. XL, LT, and XJ: paper revising. LT: paper writing. XJ: study designing. All authors contributed to the article and approved the submitted version.

## Funding

This work was supported by the Qing Lan Project and Project of Nanjing Institute of Mental Health for Minors (2021ZKWY03).

## Conflict of interest

The authors declare that the research was conducted in the absence of any commercial or financial relationships that could be construed as a potential conflict of interest.

## Publisher's note

All claims expressed in this article are solely those of the authors and do not necessarily represent those of their affiliated organizations, or those of the publisher, the editors and the reviewers. Any product that may be evaluated in this article, or claim that may be made by its manufacturer, is not guaranteed or endorsed by the publisher.
